# Gene function in schistosomes: recent advances toward a cure

**DOI:** 10.3389/fgene.2015.00144

**Published:** 2015-04-15

**Authors:** Arnon D. Jurberg, Paul J. Brindley

**Affiliations:** ^1^Laboratory on Thymus Research, Oswaldo Cruz Institute, Oswaldo Cruz Foundation/FiocruzRio de Janeiro, Brazil; ^2^Graduate Program in Cell and Developmental Biology, Institute of Biomedical Sciences, Federal University of Rio de JaneiroRio de Janeiro, Brazil; ^3^Department of Microbiology, Immunology, and Tropical Medicine, Research Center for Neglected Diseases of Poverty, School of Medicine and Health SciencesWashington, DC, USA

**Keywords:** schistosomiasis, economical impact, poverty, treatment, gene function

Neglected Tropical Diseases (NTDs) drive poverty and social inequality; indeed NTDs are manifestations of these problems (Bardosh, 2014). Poverty is a major detriment to human development. Measuring poverty by estimating poverty lines has led the World Bank to initially set the income value of US$1 a day and later update it to US$1.25 a day at 2005 purchasing power parity as the revised international poverty line (Ravallion et al., 1991, 2009). This meant that in 2005 people earning less than US$37.5 a month lived in poverty—about 1.4 billion people worldwide (Chen and Ravallion, 2008). Despite considerable efforts and highly uneven temporal and regional improvements since that point (Chen and Ravallion, 2008; United Nations, 2013), each one of these impoverished persons is potentially exposed to infectious diseases due to precarious conditions, such as contaminated drinking water, malnutrition, lack of sanitation or shelter, and inadequate basic services including education, health and security (WHO-World Health Organization, 2012; United Nations, 2013).

However, because they mostly impact populations of lower-income countries, the NTDs receive relatively little attention and funding from the international community. For example, whereas helminth infections are estimated to affect more than one billion people in sub-Saharan Africa, Asia and the Americas (Hotez et al., 2007), the global expenditures on helminth infection targeted research and development (R&D) was a mere US$66.8 million in 2008, with uneven distribution across diverse diseases and focus mainly for basic research (Institute of Medicine, 2011). In comparison, Kanavos et al. (2010) have estimated a global investment of more than US$17 billion on cancer R&D in 2007, with estimates for 2008 of more than 12.7 million new cases and ~7.6 million deaths (Ferlay et al., 2010). Although, deaths due to helminth infection, estimated to be around 300,000 deaths a year, are not as imposing as for some other infectious diseases associated mortality, helminth infection-induced burden of disease results in premature and ongoing disability and impediments throughout life, and impacts negatively and largely on the economy of endemic regions (Hotez et al., 2006). Hence, these characteristics led to the grouping of 17 parasitic, bacterial and viral diseases as NTDs by the World Health Organization (Salaam-Blyther, 2014).

Schistosomiasis is one of these NTDs, and moreover is considered the most important of the helminth diseases in terms of morbidity and mortality (Hotez et al., 2008; King, 2015). Among the most problematic of the NTDs due to its extensive geographical distribution and public health impact, schistosomiasis is caused by infection with trematode worms of the genus *Schistosoma*. Infection occurs in contaminated freshwater through skin penetration of a larval form of the worm known as the cercaria. Cercariae are produced by clonal expansion of germinal cells in the intermediate snail host following infection of the snail by a ciliated larva termed the miracidium (Pan, 1965; Cheng and Bier, 1972; Jurberg et al., 2008). There are species-specific, geographical constraints among the schistosome and the intermediate host. *Schistosoma mansoni* infects snails of the genus *Biomphalaria*, whereas *S. japonicum* and *S. haematobium* infect the genera *Oncomelania* and *Bulinus*, respectively. In these snails, the invading miracidium first transforms into mother-sporocyst then into daughter-sporocysts prior to generation of cercariae, which escape from the snail back to the freshwater. Cercariae actively swim toward a human host where they penetrate the skin directly (Pan, 1965). Within the human skin, the cercaria sheds its tail, transforms into a blood vessel inhabiting form termed the schistosomulum, which develops within the bloodstream or to a lesser extent the lymphatic system (Miller and Wilson, 1978). These schistosomula circulate with the blood as they develop to sexually mature blood flukes, sometimes completing two or three systemic circuits until they establish residency in the portal hepatic tract (*S. mansoni* and *S. japonicum*) or the pelvic organs (*S. haematobium*) (Wilson, 2009). Unlike other platyhelminths, schistosomes evolved dioecism—separate sexes—and need to mate for producing eggs. Eggs are released into the bloodstream and many embolize in smaller blood vessels and capillaries of diverse organs, inducing the characteristic granulomatous reaction of schistosomiasis (see Hams et al., 2013). Eggs reaching the intestines (*S. mansoni* and *S. japonicum*) or the bladder (*S. haematobium*) induce inflammatory responses that facilitate passage of the schistosome egg through the wall of bowel or bladder, respectively, to the lumen from where the eggs exit to the external environment in feces or urine (Lenzi et al., 1987 for *S. mansoni*). On the reaching freshwater, the egg hatches to release the motile, ciliated miracidium, thereby completing the developmental cycle of the pathogen, as depicted in Figure 1. Eggs that fail to be eliminated may cause different symptoms depending on the schistosome species, but frequently cause chronic inflammation and fibrosis (Gryseels et al., 2006; Hams et al., 2013; Colley and Secor, 2014).

**Figure 1:**
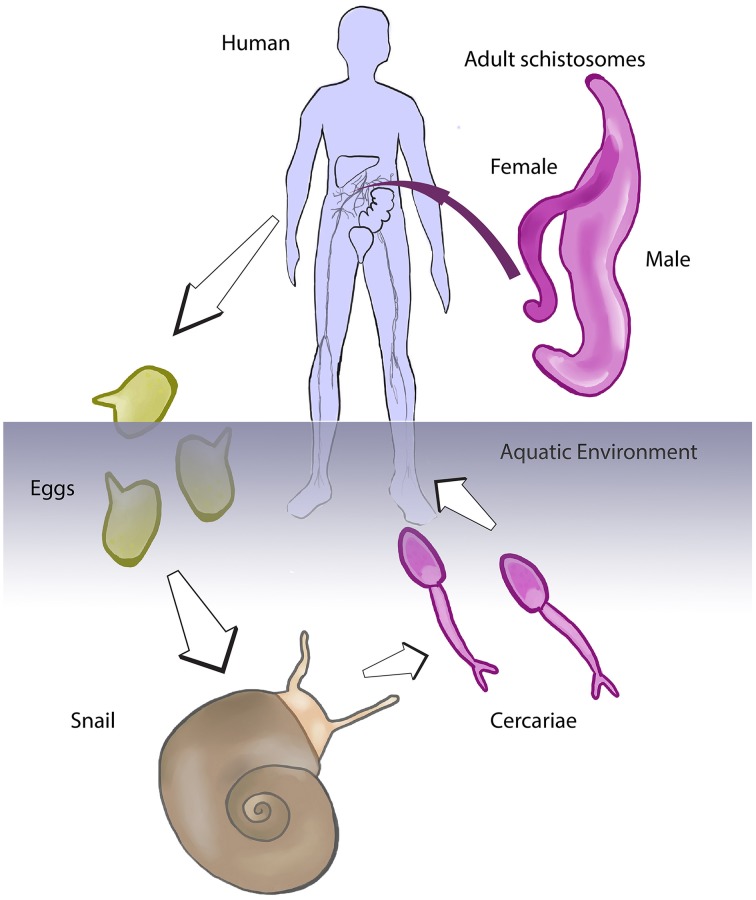
**Developmental cycle of the schistosome**. Schistosomes are obligate parasites that evolved a complex developmental cycle using two hosts, a snail and a human (or other mammal). In the human (or laboratory mouse, or other permissive mammalian host), the worms reside within the blood and reproduce sexually. Eggs released by the female schistosome exit to the external environment with the feces or urine. The egg hatches in freshwater, releasing the miracidium larva that infected the aquatic snail. The parasite undergoes asexual reproduction in the snail, producing cercariae that are shed from the snail into the water. Cercariae penetrate the skin of the human in contact with contaminated water, to complete the developmental cycle of the pathogen (we acknowledge Figure 1 of Gryseels et al. (2006) for inspiration for this schematic).

In spite of the information on schistosome biology at present, with 14,933 published papers from 1980 to 2014 (using “*Schistosoma*” as the query at PubMed)—which accounts for 78% of all the retrieved schistosome literature at this database—a definitive cure for schistosomiasis still faces daunting challenges and may be far from being achieved. In particular, 71% of the research funds for schistosomiasis in 2008 (approximately US$14 million) were designed to basic instead of applied investigation (Institute of Medicine, 2011). Yet, concurrent presence of schistosomes at different stages of development floods the infected person with a battery of diverse antigens, which induce conflicting immune responses (Colley and Secor, 2014). Moreover, naturally occurring re-infections raise the possibility that immunization against schistosomiasis may not work (Colley and Secor, 2014). Another aspect of controlling schistosome infection regards education and sanitation, once informed endemic populations still insist on, and/or frequently have no alternative but using the contaminated watercourses for bathing, laundry and other household activities, recreation, and so forth (Enk et al., 2010) (Supplementary Figure 1). Indeed, the sustained and repeated use of praziquantel in endemic regions raises the justifiable worry for the appearance of drug resistance (Cioli et al., 2014). To raise hopes for fighting schistosomiasis, game-changing advances and tools from other fields of research are being adapted and implemented for research on schistosomes, especially the strategies related to the study of gene function (Hoffmann et al., 2014). For example, deployment of the clustered regulatory interspaced short palindromic repeats (CRISPR)/Cas system for genome editing (see Doudna and Charpentier, 2014) and/or schistosome transgenesis for gain-of-function manipulation (Mann et al., 2014) extends the range of experimental approaches to interrogate the host-parasite relationship and to test novel vaccines and other interventions. It is now the time for research on schistosomiasis to evolve from -omics to function.

Our new research topic includes 15 papers, both primary research articles and reviews from a representative cadre of the leading experts in the field of research on schistosomes and schistosomiasis, and neglected tropical diseases. The themes span molecular genetics including chromosomal evolution, epigenetic control of schistosome genes, vaccine studies including targeting proteolysis and enzyme inhibitors central to the physiology of the parasites, and how infection with this NTD pathogen induces bladder cancer. The reports also address signaling pathways, including insulin receptors in these pathogens, kinomes, and kinases, glycogenome, molecular studies on sex differentiation and host-parasite interactions including the snail-schistosome relationship. The 91 authors are from 14 countries, including from regions where schistosomiasis is endemic such as Angola, Egypt, Brazil, and China. We hope you will be informed by this series, as well as enjoy the authors' scholarly contributions, that the work and ideas presented advance the field toward better control or even a cure for schistosomiasis, and that this Research Topic elevates investigation in schistosomiasis and related NTDs into the more newsworthy areas of emerging and infectious disease research.

## Conflict of interest statement

The authors declare that the research was conducted in the absence of any commercial or financial relationships that could be construed as a potential conflict of interest.

## Supplementary material

Supplementary Figure 1 **A representative endemic region for schistosomiasis, in this example in rural Minas Gerais state, Brazil**. The triptych illustrates the role of sub-standard sanitation and of human activities in contaminated watercourses that also are ecosystems that include the snail intermediate hosts of schistosomiasis (Photographs kindly provided by Áureo Almeida and Paulo Marcos Coelho, René Rachou Research Center/Fiocruz-MG, Brazil).
