# Supplementary figures and images for: Gene function in schistosomes: recent advances toward a cure
Source: Front Genet. 2015 Apr 15;6:144. doi: 10.3389/fgene.2015.00144 (PMC4397921; doi:10.3389/fgene.2015.00144)

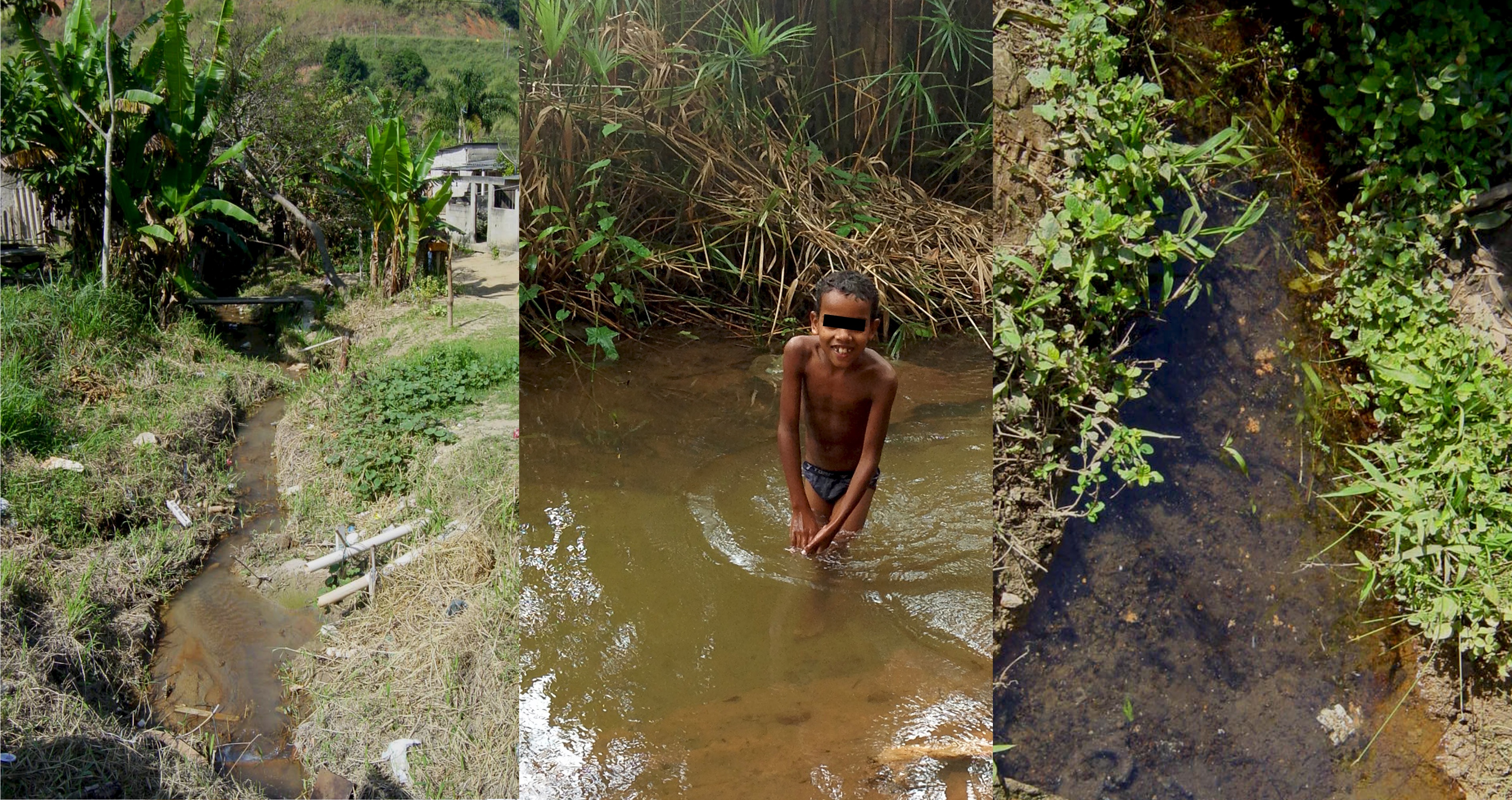

Supplement: Supplementary Figure 1 — A representative endemic region for schistosomiasis, in this example in rural Minas Gerais state, Brazil. The triptych illustrates the role of sub-standard sanitation and of human activities in contaminated watercourses that also are ecosystems that include the snail intermediate hosts of schistosomiasis (Photographs kindly provided by Áureo Almeida and Paulo Marcos Coelho, René Rachou Research Center/Fiocruz-MG, Brazil). [file Image1.JPEG]
